# Empagliflozin Attenuates Liver Inflammation and Fibrosis in NAFLD: Evidence from Mendelian Randomization and Mouse Experiments

**DOI:** 10.3390/cimb47100846

**Published:** 2025-10-15

**Authors:** Chao Fu, Lijiao Deng, Xiaochan Zhu, Bin Wang, Bin Hu, Huan Xue, Qingxuan Zeng, Yi Zhang

**Affiliations:** 1 Department of Pharmacology, School of Basic Medicine, Shanxi Medical University, Taiyuan 030001, China; fuchao@sxmu.edu.cn (C.F.); dlj18735088748@163.com (L.D.); zxc2556674278@163.com (X.Z.); wangbin41@sxmu.edu.cn (B.W.); hubinnihao1223@sxmu.edu.cn (B.H.); xuehuan825167483@126.com (H.X.); zengqingxuan@sxmu.edu.cn (Q.Z.); 2 School of Pharmacy, Shanxi Medical University, Taiyuan 030001, China; 3 Medicinal Basic Research Innovation Center of Chronic Kidney Disease, Ministry of Education, Shanxi Medical University, Taiyuan 030001, China

**Keywords:** non-alcoholic fatty liver disease, empagliflozin, SGLT2 inhibitor, NF-κB, inflammation, fibrosis, α-SMA, COL1A1, Mendelian randomization

## Abstract

Non-alcoholic fatty liver disease (NAFLD) is a prevalent chronic liver disorder and a major global health challenge, yet effective pharmacological therapies are lacking. Empagliflozin, a sodium–glucose cotransporter-2 (SGLT2) inhibitor, has shown systemic metabolic and anti-inflammatory benefits, but its liver-specific molecular mechanisms remain incompletely understood. In this study, we evaluated the therapeutic effects of empagliflozin in a diet-induced mouse model of NAFLD, supported by Mendelian randomization analysis. Histological examination, serum biochemistry, and hepatic triglyceride quantification demonstrated that empagliflozin markedly attenuated hepatic steatosis and improved liver injury indices. At the molecular level, empagliflozin suppressed NF-κB-mediated inflammatory signaling and significantly downregulated fibrotic markers including α-SMA and COL1A1, while modulating TIMP-1 and MMP-9 expression. Collectively, these findings reveal that empagliflozin ameliorates NAFLD by inhibiting inflammatory and fibrotic molecular pathways, highlighting its potential as a mechanism-based therapeutic option for NAFLD.

## 1. Introduction

Non-alcoholic fatty liver disease (NAFLD), recently redefined as metabolic dysfunction-associated steatotic liver disease (MASLD) [1], represents the most prevalent chronic liver disorder, affecting nearly one-quarter of the global population [2,3]. The revised terminology highlights the central role of metabolic dysfunction in disease pathogenesis; nevertheless, the designation “NAFLD” continues to be extensively used in basic and translational research and is therefore adopted herein for consistency with prior studies [4]. The pathological continuum of NAFLD extends from simple steatosis to non-alcoholic steatohepatitis (NASH) [5], which may evolve into progressive fibrosis, cirrhosis, and hepatocellular carcinoma [6,7]. The global rise in NAFLD prevalence parallels epidemics of obesity, type 2 diabetes mellitus, and metabolic syndrome, rendering it a major health and socioeconomic challenge [8]. Lifestyle modification remains the mainstay of management [9]; however, long-term adherence is notoriously difficult. Although resmetirom (2024) and semaglutide/Wegovy (2025) have recently received accelerated approval for MASH with moderate-to-advanced fibrosis [9,10], effective and broadly applicable pharmacotherapies for the wider NAFLD population remain elusive [11]. This therapeutic gap underscores the urgency of developing mechanism-based interventions targeting key molecular drivers of disease progression [12].

The pathogenesis of NAFLD is orchestrated by a complex interplay of lipid accumulation, lipotoxicity, oxidative stress, inflammatory signaling, and extracellular matrix (ECM) remodeling [13,14,15]. Hepatic steatosis perturbs mitochondrial homeostasis and activates nuclear factor kappa B (NF-κB), thereby amplifying inflammatory cascades and inducing the release of cytokines such as tumor necrosis factor-α (TNF-α), interleukin-6 (IL-6), and interleukin-1β (IL-1β) [16]. Sustained activation of these pathways promotes hepatocellular damage and immune cell infiltration. In parallel, chronic inflammation stimulates hepatic stellate cell activation and drives fibrogenic remodeling characterized by upregulation of α-smooth muscle actin (α-SMA), collagen type I alpha 1 (COL1A1), tissue inhibitor of metalloproteinases-1 (TIMP-1), and matrix metalloproteinase-9 (MMP-9), culminating in ECM deposition and fibrosis [17,18]. These processes collectively constitute the molecular hallmarks of NAFLD progression and provide tractable targets for therapeutic intervention.

Sodium–glucose cotransporter-2 (SGLT2) inhibitors have emerged as a cornerstone in the treatment of type 2 diabetes mellitus, with robust evidence of additional benefits on cardiovascular and renal outcomes [19]. Beyond systemic metabolic regulation, accumulating studies indicate that SGLT2 inhibition may also confer hepatoprotective effects by ameliorating insulin resistance [20], attenuating hepatic steatosis, and modulating inflammatory pathways [21]. Empagliflozin, a prototypical SGLT2 inhibitor, has been increasingly documented to improve metabolic and hepatic outcomes in both preclinical and clinical settings [22]. Nonetheless, the precise molecular mechanisms underlying its protective effects in NAFLD, particularly concerning inflammatory and fibrotic signaling, remain insufficiently defined [23].

To establish causality and strengthen the rationale for mechanistic investigation, Mendelian randomization (MR) has been widely applied to leverage genetic variants as instrumental variables linking exposures to disease traits. In the present study, we first employed MR to evaluate the potential causal relationship between SGLT2-related genetic determinants and NAFLD susceptibility. We subsequently validated these associations in a diet-induced murine model, integrating histological, biochemical, and molecular analyses. This combined approach aimed to delineate the molecular underpinnings of empagliflozin’s hepatoprotective actions and to provide translational insights into its therapeutic potential for NAFLD.

## 2. Materials and Methods

### 2.1. Animals and Experimental Design

Male C57BL/6J mice were used as normal controls (NC). Leptin-deficient ob/ob mice were purchased from Cavens Laboratory (Changzhou, China) and randomly allocated into three groups (*n* = 6 per group): (1) model group, receiving GAN diet only; (2) semaglutide-treated group (SEMA), receiving GAN diet plus semaglutide; and (3) empagliflozin-treated group (EMP), receiving GAN diet plus empagliflozin. NC mice were maintained on standard chow, whereas all other groups were fed a GAN diet containing 40% fat, 20% fructose, and 2% cholesterol [24] (Jiangsu Xietong Pharmaceutical Bioengineering Co., Ltd., Nanjing, China). Semaglutide was administered subcutaneously at a dose of 20 nmol/kg/day, while empagliflozin was administered by oral gavage at 8 mg/kg/day; model mice received vehicle only. Interventions lasted for 5 weeks. Tail-vein blood was collected at week 3 for serum biochemistry. At week 5, all mice were euthanized, and blood and liver tissues were harvested for subsequent histological, biochemical, and molecular analyses. All mice were maintained in a controlled environment at a temperature of 22 ± 1 °C, relative humidity of 50 ± 5%, and a 12:12 h light/dark cycle, with ad libitum access to food and water. This study was approved by the Institutional Animal Care and Use Committee (IACUC) of Shanxi Medical University and conducted in accordance with the Guide for the Care and Use of Laboratory Animals (approval number: SYXK IACUC 2024-0007).

### 2.2. Biochemical Analyses

Blood samples were collected from the tail vein at week 3 and from the retro-orbital plexus at the time of sacrifice (week 5). Serum was separated by centrifugation at 3000× *g* for 10 min at 4 °C and stored at −80 °C until analysis. Serum alanine aminotransferase (ALT), aspartate aminotransferase (AST), triglycerides (TG), and total cholesterol (TC) were measured using commercial kits (Kubel Biotechnology, Shenzhen, China) according to the manufacturers’ instructions. Hepatic TC, TG, malondialdehyde (MDA), and reduced glutathione (GSH) levels were determined using assay kits purchased from Solarbio (Beijing, China). All assays were performed in duplicate to ensure reproducibility.

### 2.3. Histological Examination

Liver tissues were fixed in 4% paraformaldehyde, embedded in paraffin, and sectioned at a thickness of 4 μm. Sections were stained with hematoxylin and eosin (H&E) to evaluate hepatic steatosis, inflammation, and hepatocellular injury. Fibrosis was assessed by Sirius Red staining of paraffin-embedded sections. Stained slides were examined under a light microscope (Eclipse Ci-L, Nikon, Tokyo, Japan), and representative images were captured. Histological evaluation was independently performed by two investigators blinded to group allocation.

### 2.4. Gene Expression Analysis

Total RNA was isolated from frozen liver tissues using Monzol™ Reagent Pro (Monad Biotech, Wuhan, China) according to the manufacturer’s instructions. cDNA was synthesized from 1 μg of RNA using ToloScript RT EasyMix (ToloBio, Shanghai, China). Quantitative real-time PCR (qPCR) was performed using 2× M5 HiPer SYBR Premix EsTaq (Mei5Bio, Beijing, China) on a StepOnePlus Real-Time PCR System (Applied Biosystems, Foster City, CA, USA). Target genes included sterol regulatory element-binding protein-1c (SREBP-1c), fatty acid synthase (FASN), tumor necrosis factor-α (TNF-α), interleukin-1β (IL-1β), interleukin-6 (IL-6), nuclear factor kappa B (NF-κB), alpha-smooth muscle actin (α-SMA), collagen type I alpha 1 (COL1A1), tissue inhibitor of metalloproteinases-1 (TIMP-1), and matrix metalloproteinase-9 (MMP-9). Expression levels were normalized to glyceraldehyde-3-phosphate dehydrogenase (GAPDH) as an internal control, and relative expression was calculated using the 2^−ΔΔCt^ method. Primer sequences are listed in Table 1.

### 2.5. Mendelian Randomization Analysis

Genetic proxies for SGLT2 inhibition were selected using variants associated with glycated hemoglobin (HbA1c), as SGLT2 inhibitors have consistently been shown to reduce HbA1c in randomized controlled trials of type 2 diabetes [25,26,27]. We chose HbA1c as the biomarker of SGLT2 action because of the established effect of SGLT2 inhibition on this marker of glycaemic control [28]. To instrument SGLT2, we selected missense (protein coding) variants within the SLC5A2 gene (build GRCh37/hg19: chromosome 16: 31494444–31502090) that were associated with HbA1c at genome-wide significance (*p* < 5 × 10^−8^) and uncorrelated (linkage disequilibrium threshold of r^2^ < 0.3 using PLINK and phase 3 version 5 of the 1000 genomes project as reference panel). The HbA1c GWAS comprised 389,889 participants of European ancestry in the UK Biobank [29]. The SNPs within 100 kilobases of the drug target, with a genome-wide significance *p* less than 5 × 10^−8^ and a linkage disequilibrium R^2^ less than 0.3, were used as genetic instruments [30].

Genetic association for outcomes: Fibrosis and cirrhosis of liver, 2653 samples of disease and 485,213 samples of control. Dataset access: https://storage.googleapis.com/finngen-public-data-r12/summary_stats/release/finngen_R12_K11_FIBROCHIRLIV.gz (accessed on 15 January 2025)

Statistical analysis and MR assumptions: Analysis using multiple instruments for genetically predicted HbA1c was performed using the inverse-variance weighted (IVW) method, which provides a weighted average of variant estimates analogous to a fixed-effect meta-analysis [31]. In the meanwhile, pleiotropy and heterogeneity test statistics (Cochran Q-derived p) were calculated using the IVW, MR-Egger, and MR-Pleiotropy Residual Sum and Outlier methods (MR-PRESSO) [32]. Pleiotropy and heterogeneity were regarded as nonexistent when the *p*-value was higher than 0.05. IVW was used as the primary method in the absence of pleiotropy and heterogeneity; otherwise, MR-Egger was used. Finally, we utilized leave-one-out analysis to see if a single SNP was the source of the MR estimate bias. The analyses were carried out using the R packages TwoSampleMR (version 0.5.6) and MendelR (version 2.1.2). All analyses were carried out in R (version 4.0) [33].

### 2.6. Statistical Analysis

Data are expressed as mean ± SEM. Multi-group comparisons were performed using one-way ANOVA with Tukey’s post hoc test. Repeated measures (e.g., body weight) were analyzed with two-way ANOVA. *p* < 0.05 was considered statistically significant (* *p* < 0.05, ** *p* < 0.01, *** *p* < 0.001 vs. model). Analyses and graphs were generated with GraphPad Prism v9.0 (GraphPad Software, Boston, MA, USA).

## 3. Results

### 3.1. Genetic Evidence Supporting SGLT2 Inhibition in NAFLD

**Figure 1:**
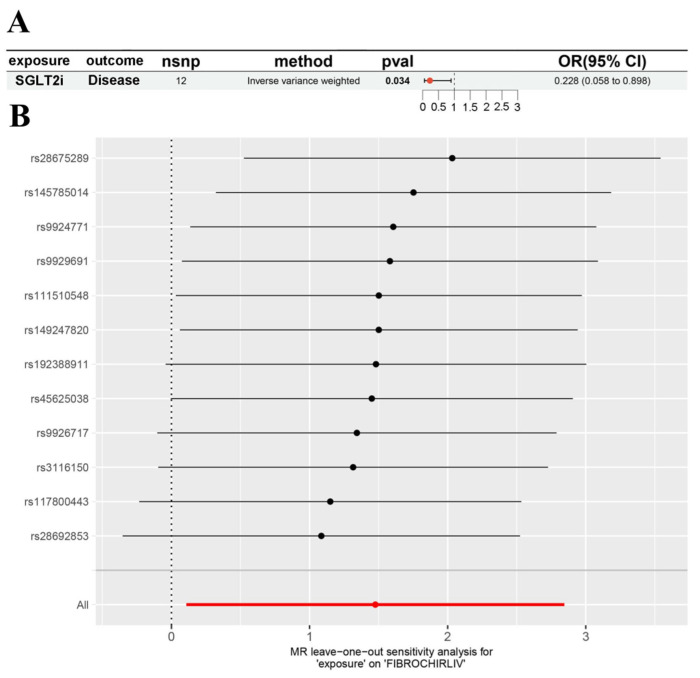
Mendelian randomization (MR) analysis of SGLT2 inhibition and liver fibrosis. (**A**) Causal effect estimate of genetically proxied SGLT2 inhibition (HbA1c-associated variants in the SLC5A2 locus) on liver fibrosis/cirrhosis using FinnGen data. (**B**) Leave-one-out sensitivity analysis showing that the overall causal estimate was not driven by any single SNP. No evidence of heterogeneity or horizontal pleiotropy was observed.

Mendelian randomization (MR) was performed using single-nucleotide polymorphisms (SNPs) within the SLC5A2 locus as genetic instruments to evaluate the causal effect of SGLT2 inhibition on liver fibrosis and cirrhosis. Inverse variance weighted (IVW) estimation indicated that genetically proxied SGLT2 inhibition was significantly associated with a reduced risk of liver fibrosis/cirrhosis (Figure 1A). Sensitivity analyses, including weighted median and MR-Egger regression, yielded consistent results with no evidence of substantial heterogeneity or horizontal pleiotropy (Figure 1B). Instrument strength was confirmed, with all F-statistics > 10 (Table 2). Collectively, these findings support a protective role of SGLT2 inhibition against liver fibrosis.

### 3.2. Empagliflozin Alleviates Metabolic Burden and Liver Injury

**Figure 2:**
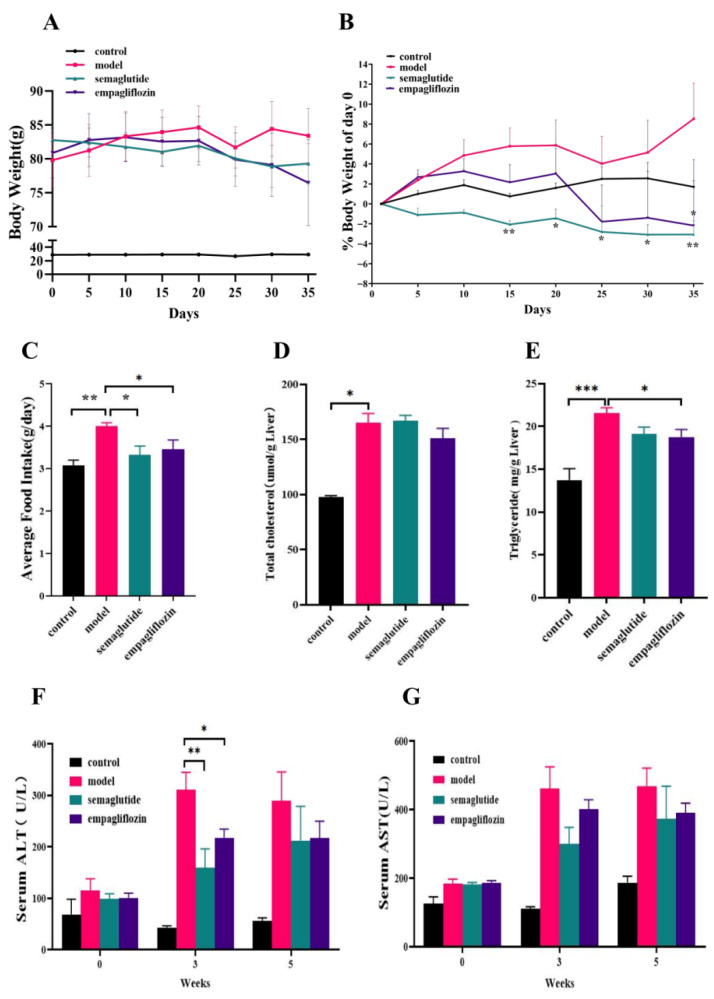
Empagliflozin improves metabolic and liver function phenotypes in ob/ob mice fed with the GAN diet. (**A**) Body weight trajectory of mice during the 5-week treatment. (**B**) Percent change in body weight relative to day 0. (**C**) Average daily food intake during the experiment. (**D**) Hepatic total cholesterol (TC) levels at the end of the experiment. (**E**) Hepatic triglyceride (TG) levels at the end of the experiment. (**F**) Serum alanine aminotransferase (ALT) levels at weeks 3 and 5. (**G**) Serum aspartate aminotransferase (AST) levels at weeks 3 and 5. Data are presented as mean ± SEM (*n* = 6 per group). * *p* < 0.05, ** p < 0.01, *** *p* < 0.001.

Excessive weight gain and hepatic lipid accumulation are hallmarks of NAFLD pathogenesis and progression. To evaluate the effects of empagliflozin (EMP), we assessed body weight, food intake, and hepatic lipid content in mice. Compared with the normal control (NC) group, GAN-fed ob/ob mice (model group) exhibited significant increases in body weight, whereas EMP treatment significantly reduced body weight and percent body weight change (Figure 2A,B). Food intake was significantly higher in the model group compared with NC, while EMP partially reduced food intake, although this reduction did not fully account for the observed weight loss (Figure 2C). Hepatic lipid analysis revealed that both total cholesterol (TC) and triglyceride (TG) levels were significantly higher in the model group compared with controls. For hepatic TC, EMP treatment showed a downward trend without reaching statistical significance (Figure 2D). In contrast, EMP significantly decreased hepatic TG levels (Figure 2E). In addition, serum alanine aminotransferase (ALT) and aspartate aminotransferase (AST) levels were markedly increased in the model group, and EMP treatment ameliorated these elevations (Figure 2F,G).

### 3.3. Empagliflozin Ameliorates Hepatic Steatosis and Inflammatory Responses

**Figure 3:**
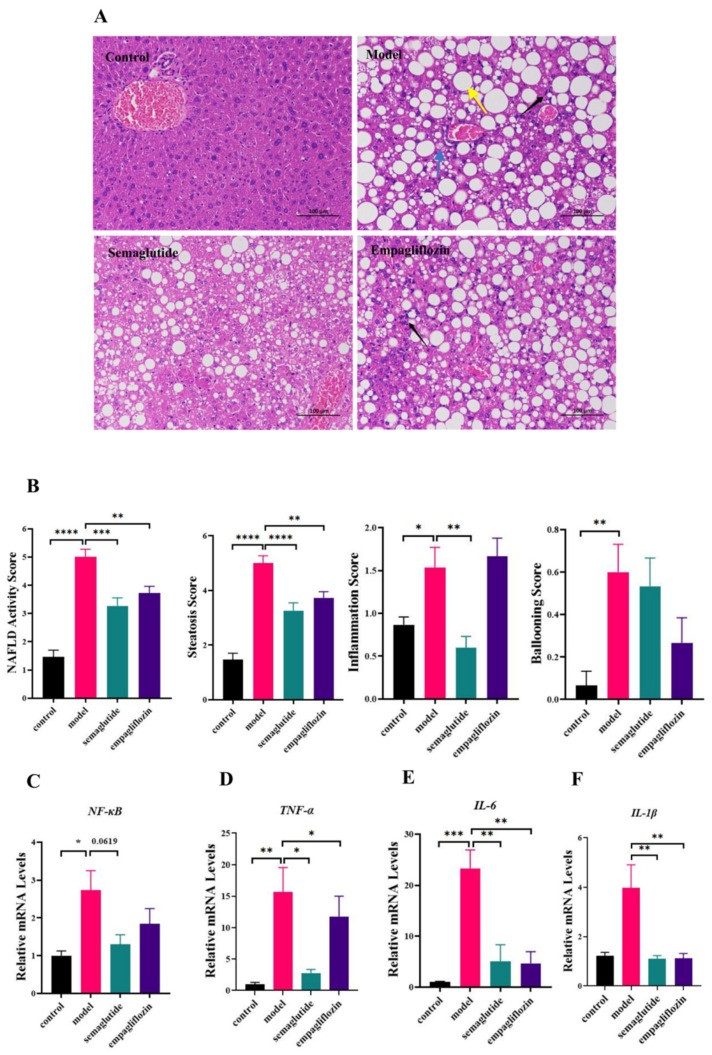
Empagliflozin improves hepatic steatosis and inflammatory responses in NAFLD mice. (**A**) Representative histology of H&E staining (scale bar = 100 μm), Colored arrows: yellow—lipid droplets; blue—ballooning; black—lobular inflammation. (**B**) Semi-quantitative analysis of steatosis, inflammation, ballooning, and the composite NAFLD activity score (NAS). (**C**–**F**) Hepatic mRNA expression levels of NF-κB, TNF-α, IL-6, and IL-1β, normalized to GAPDH. Data are presented as mean ± SEM. * *p* < 0.05, ** *p* < 0.01, *** *p* < 0.001, **** *p* < 0.0001.

H&E staining showed abundant lipid droplet accumulation (yellow arrows), ballooning degeneration (blue arrows), and lobular inflammation (black arrows) in GAN-fed ob/ob mice. These histological abnormalities were markedly alleviated in the empagliflozin group, similar to the effects of semaglutide (Figure 3A). Semi-quantitative analysis indicated that the NAS and its subscores (steatosis, inflammation, ballooning) were significantly elevated in the model group. EMP significantly reduced the steatosis score, while ballooning degeneration exhibited a downward trend without reaching statistical significance (Figure 3B). At the transcriptional level, NF-κB mRNA expression was significantly upregulated in the model group, whereas empagliflozin (EMP) treatment resulted in a non-significant reduction in NF-κB mRNA levels (Figure 3C). In contrast, TNF-α, IL-6, and IL-1β expression levels were significantly downregulated by EMP treatment (Figure 3D–F).

### 3.4. Empagliflozin Regulates Lipid Metabolism and Oxidative Stress in NAFLD Mice

**Figure 4:**
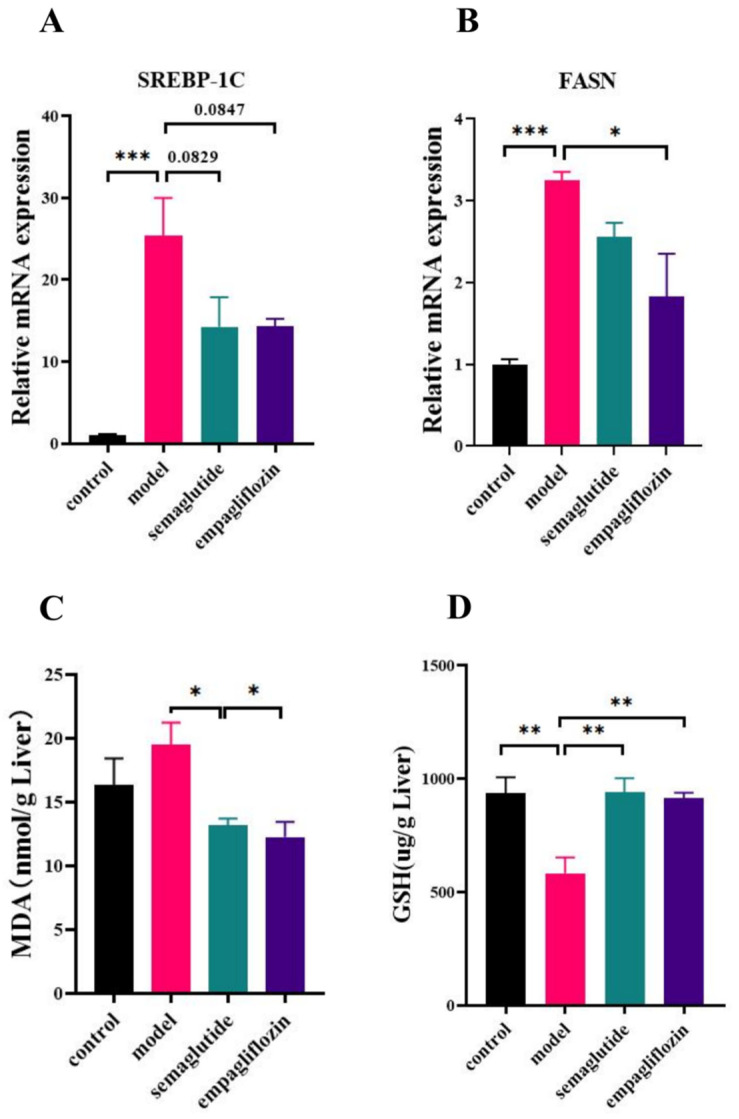
Empagliflozin regulates hepatic lipid metabolism and oxidative stress in ob/ob mice fed with the GAN diet. (**A**) Hepatic mRNA expression of SREBP-1c. (**B**) Hepatic mRNA expression of FASN. (**C**) Hepatic malondialdehyde (MDA) levels. (**D**) Hepatic reduced glutathione (GSH) levels. Data are presented as mean ± SEM. * *p* < 0.05, ** *p* < 0.01, *** *p* < 0.001.

Hepatic expression of lipogenic genes was markedly upregulated in the model group compared with controls. SREBP-1c expression was strongly increased, and empagliflozin (EMP) treatment showed a downward trend that did not reach statistical significance (Figure 4A). In contrast, FASN expression was significantly suppressed by EMP compared with the model group (Figure 4B). Regarding oxidative stress, hepatic MDA levels were significantly elevated in the model group, and EMP treatment significantly decreased MDA levels (Figure 4C). Meanwhile, hepatic GSH content was markedly reduced in the model group, whereas EMP significantly restored GSH levels toward normal (Figure 4D).

### 3.5. Empagliflozin Attenuates Hepatic Fibrosis in NAFLD Mice

**Figure 5:**
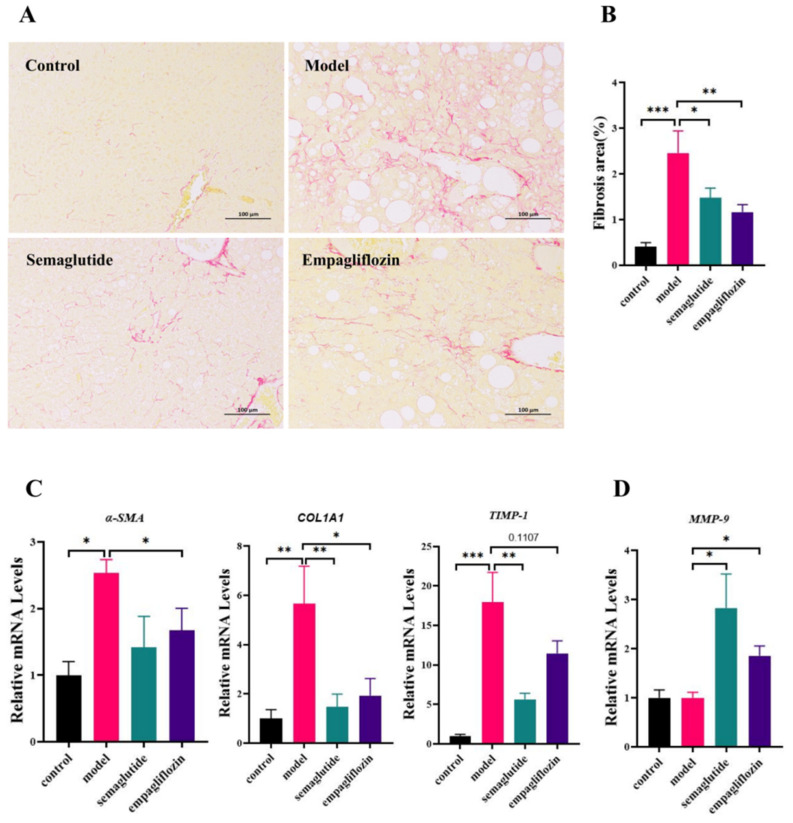
Empagliflozin attenuates hepatic fibrosis in ob/ob mice fed with the GAN diet. (**A**) Sirius Red staining of liver sections (scale bar = 100 μm). (**B**) Quantification of fibrotic area (%). (**C**) Hepatic mRNA expression of α-SMA, COL1A1, and TIMP-1. (**D**) Hepatic mRNA expression of MMP-9. Data are presented as mean ± SEM. * *p* < 0.05, ** p < 0.01; *** *p* < 0.001.

Liver fibrosis is a key pathological feature driving the progression of NAFLD. Sirius Red staining revealed extensive collagen deposition in the model group, whereas empagliflozin (EMP) treatment markedly reduced fibrotic accumulation (Figure 5A). Quantitative analysis further confirmed a significant increase in fibrotic area in the model group, which was significantly decreased by EMP treatment (Figure 5B). At the molecular level, EMP exerted a pronounced antifibrotic effect by significantly downregulating α-SMA and COL1A1, two hallmark markers of hepatic stellate cell activation and extracellular matrix deposition (Figure 5C). In contrast, EMP had no significant effect on TIMP-1 expression (Figure 5C). Notably, MMP-9 expression was markedly reduced in the model group, and EMP treatment significantly restored its levels, indicating enhanced matrix degradation and remodeling capacity (Figure 5D).

## 4. Discussion

In this study, we systematically evaluated the role and potential mechanisms of the SGLT2 inhibitor empagliflozin in NAFLD by integrating Mendelian randomization (MR) analysis with in vivo experiments. MR analysis demonstrated a significant protective association between genetically proxied SGLT2 inhibition and reduced risk of liver fibrosis/cirrhosis, providing genetic evidence for causality and translational relevance. In the NAFLD mouse model, empagliflozin markedly alleviated body weight gain and hepatic lipid accumulation, improved histological injury, suppressed lipogenesis, mitigated oxidative stress, and downregulated inflammatory and fibrosis-related gene expression. These effects suggesting that empagliflozin exerts beneficial effects on NAFLD progression through multiple mechanisms. To our knowledge, this is the first study to provide dual validation at both the genetic and experimental levels, strengthening the rationale for empagliflozin as a potential therapeutic option for NAFLD.

In recent years, the therapeutic potential of SGLT2 inhibitors in metabolic diseases has gained increasing attention, with emerging evidence suggesting benefits in NAFLD [34,35]. Previous studies have reported that empagliflozin improves hepatic lipid metabolism indirectly by reducing body weight, glucose levels, and insulin resistance [36]. Experimental studies in animal models have also demonstrated that SGLT2 inhibitors attenuate hepatic steatosis and inflammatory responses [37,38]. Our findings are consistent with these observations but extend them by providing both genetic evidence from Mendelian randomization and experimental validation, thereby reinforcing causality and translational significance. Moreover, we observed that empagliflozin exerted regulatory effects on inflammatory cytokines and fibrosis-related genes comparable to those of the GLP-1 receptor agonist semaglutide, which aligns with previous reports highlighting the hepatoprotective actions of GLP-1RAs in NAFLD [39]. Compared with thiazolidinediones such as pioglitazone, empagliflozin may offer additional benefits in terms of body weight control and oxidative stress reduction. Taken together, our study not only confirms prior observational findings on SGLT2 inhibitors but also extends mechanistic evidence to the inflammatory and fibrotic pathways in NAFLD [40].

Our findings suggest that empagliflozin exerts protective effects against NAFLD through multiple mechanisms. Although empagliflozin partially reduced food intake in our study, this modest change alone cannot account for the marked decrease in body weight. SGLT2 inhibitors promote urinary glucose excretion, causing an energy loss of about 200–300 kcal per day, and enhance insulin sensitivity and lipid oxidation, thereby improving metabolic efficiency [27]. Consistent with these mechanisms, EMP downregulated hepatic FASN expression and oxidative stress markers, indicating that its weight-lowering effect mainly results from enhanced energy expenditure and reduced lipogenesis rather than simple caloric restriction [37]. Collectively, these data indicate that the reduction in body weight induced by EMP reflects metabolic reprogramming rather than decreased appetite. First, Mendelian randomization analysis revealed a significant protective association between SGLT2 inhibition and liver fibrosis, providing causal evidence that supports clinical translation. Together with animal experiments, this indicates that metabolic improvement is a fundamental mechanism underlying the beneficial effects of SGLT2 inhibition in NAFLD. Second, empagliflozin markedly suppressed hepatic expression of lipogenesis-related genes, including SREBP-1c and FASN, suggesting a direct role in reducing hepatic lipid synthesis and accumulation. In addition, empagliflozin decreased hepatic MDA levels and restored GSH, indicating its ability to mitigate oxidative stress, a key driver of NAFLD progression. Moreover, empagliflozin reduced the expression of NF-κB, TNF-α, IL-6, and IL-1β, supporting an anti-inflammatory effect, while simultaneously downregulating α-SMA, COL1A1, and TIMP-1 expression and restoring MMP-9, thereby exerting antifibrotic effects. Notably, many of these mechanisms were comparable to those observed with semaglutide, suggesting overlapping protective pathways between SGLT2 inhibitors and GLP-1 receptor agonists. Collectively, these results highlight the multifaceted actions of empagliflozin in regulating metabolism, lipid synthesis, oxidative stress, inflammation, and fibrosis, providing new mechanistic evidence for its role as a multi-target intervention in NAFLD [41].

At present, no specific pharmacological therapy has been approved for NAFLD, and current management primarily relies on lifestyle interventions and control of metabolic risk factors [42,43]. Thus, identifying novel agents with pleiotropic effects is of great clinical importance. By integrating genetic and experimental evidence, our study suggests that the SGLT2 inhibitor empagliflozin exerts significant benefits in reducing hepatic lipid accumulation, suppressing inflammation, and attenuating fibrosis, thereby supporting its therapeutic potential in NAFLD. Importantly, the effects of empagliflozin were comparable to those of semaglutide, which has demonstrated histological improvements in NASH in recent clinical trials [40,44], indicating that empagliflozin may represent an alternative therapeutic candidate alongside GLP-1 receptor agonists. Moreover, SGLT2 inhibitors are already widely used in the treatment of type 2 diabetes and heart failure, with well-established cardiovascular and renal benefits [45]. This makes empagliflozin particularly attractive for NAFLD patients with comorbid metabolic syndrome and cardiovascular disease, where dual benefits may be achieved. Future studies should also evaluate the potential of combination therapies, such as empagliflozin with GLP-1 receptor agonists, to achieve synergistic effects in metabolic regulation and hepatic protection.

Although this study combined genetic and experimental evidence to comprehensively evaluate the role of empagliflozin in NAFLD, several limitations should be acknowledged. First, the MR analysis was based on GWAS data from European populations, and its generalizability to other ethnic groups remains to be established [46]. Second, the ob/ob mouse model with high-fat and high-fructose diet recapitulates certain metabolic and histological features of NAFLD but does not fully mimic the complex pathophysiology of human disease [47]. Third, the five-week intervention period in our animal study was relatively short, which may not be sufficient to fully evaluate long-term outcomes such as advanced fibrosis, cirrhosis, or hepatocellular carcinoma. Longer treatment durations will be considered in future studies [48]. Fourth, our analysis was limited to mRNA expression without protein-level validation (e.g., Western blot or immunohistochemistry), which will be included in future work to confirm these findings. Future research should therefore include MR analyses across diverse populations and prospective clinical trials to validate the causal role and therapeutic efficacy of empagliflozin in NAFLD. Moreover, combination strategies with other agents, such as GLP-1 receptor agonists, warrant investigation to achieve synergistic effects in metabolic and hepatic protection [49,50]. Finally, in-depth mechanistic studies exploring metabolic reprogramming, mitochondrial function, and immune microenvironment regulation will be essential to fully elucidate the multidimensional protective actions of SGLT2 inhibition in NAFLD [51].

By integrating genetic inference with experimental validation, this study provides comprehensive evidence supporting the protective role of the SGLT2 inhibitor empagliflozin in NAFLD. Our findings demonstrated that SGLT2 inhibition was significantly associated with a reduced risk of liver fibrosis and that empagliflozin ameliorated weight gain, hepatic lipid accumulation, histological injury, lipogenesis, oxidative stress, and the expression of inflammatory and fibrosis-related genes in NAFLD mice. These results offer important mechanistic insights and translational value for the use of empagliflozin in NAFLD. In conclusion, SGLT2 inhibitors hold promise as potential therapeutic candidates for NAFLD, although their long-term efficacy and clinical safety require further investigation.

## 5. Conclusions

In conclusion, this study provides convergent genetic and experimental evidence that empagliflozin (EMP) confers protection against NAFLD progression. Mendelian randomization demonstrated a causal protective association between SGLT2 inhibition and reduced risk of liver fibrosis/cirrhosis, while in vivo experiments revealed that EMP attenuates hepatic steatosis and liver injury, improves redox balance, and suppresses inflammatory and fibrotic responses. At the molecular level, EMP significantly downregulated lipogenic and fibrotic markers and restored antioxidant and matrix-remodeling capacity. Collectively, these findings highlight EMP as a promising mechanism-based therapeutic option for NAFLD, supporting its potential for future translational and clinical applications.

## Figures and Tables

**Table 1:** Primer sequences used for qRT-PCR (Mus musculus).

Gene	Forward (5′→3′)	Reverse (5′→3′)
GAPDH	ATG GTG AAG GTC GGT GTG AA	CGC TCC TGG AAG ATG GTG AT
SREBP1c	GGA GCC ATG GAT TGC ACA TT	CAG GAA GGC TTC CAG AGA GG
FASN	GCC TAC ACC CAG AGC TAC CG	GCC ATG GTA CTT GGC CTT G
TNF-α	ATG GAT CTC AAA GAC AAC CAA CTA G	ACG GCA GAG AGG AGG TTG ACT T
IL-1β	TCG TGC TGT CGG ACC CAT AT	GGT TCT CCT TGT ACA AAG CTC ATG
IL-6	AAC CAC GGG CTT CCC TAC TT	TCT GTT GGG AGT GGT ATC CTC TGT
NF-κB	ATG GCA GAC GAT GAT CCC TAC	CGG ATC GAA ATC CCC TCT GTT
α-SMA	GCT TCG CTG GTG ATG ATG CTC	AGT TGG TGA TGA TGC CGT GTT C
COL1a1	GAC AGG CGA ACA AGG TGA CAG AG	CAG GAG AAC CAG GAG AAC CAG GAG
TIMP-1	GCA TCT CTG GCA TCT GGC ATC C	CGC TGG TAT AAG GTG GTC TCG TTG
MMP-9	AGC ACG GCA ACG GAG AAG G	CCA CTC GGG TAG GGC AGA AG

**Table 2:** Heterogeneity and pleiotropy tests for Mendelian randomization analysis.

Outcome	Drug	NSNPs	Pleiotropy			Heterogeneity			
						MR Egger		Inverse Variance Weighted	
			Egger Intercept	SE	*p* Value	Q	*p* Value	Q	*p* Value
Fibrosis and cirrhosis of liver	SGLT2i	12	0.0513	0.0303	0.1212	8.48	0.58	11.34	0.41

## Data Availability

The original contributions presented in this study are included in the article. Further inquiries can be directed to the corresponding author(s).
